# Construct elaboration and validity of the Pregnancy Depression Risk Scale

**DOI:** 10.1590/0034-7167-2022-0306

**Published:** 2023-03-06

**Authors:** Mônica Maria de Jesus Silva, Claudia Benedita dos Santos, Maria José Clapis

**Affiliations:** I Universidade de São Paulo. Ribeirão Preto, São Paulo, Brazil

**Keywords:** Nursing, Depression, Pregnancy, Psychometrics, Validation Study., Enfermería, Depresión, Embarazo, Psicometría, Estudio de Validación., Enfermagem, Depressão, Gravidez, Psicometria, Estudo de Validação.

## Abstract

**Objectives:**

to elaborate and analyze the Pregnancy Depression Risk Scale psychometric properties.

**Methods:**

methodological research, in six steps: theoretical model empirical definition; elaboration of scale items with literature review; consultation with five professional health experts and 15 pregnant women; content validity with six experts; pre-test-semantic validity with 24 pregnant women; scale factor structure definition with 350 pregnant women; pilot study with 100 pregnant women, totaling 489 pregnant women and 11 experts. Data were analyzed by content analysis, exploratory factor analysis, multitrait-multimethod analysis and internal consistency.

**Results:**

sixty-eight risk factors were identified for item formulation. The final version of the scale consisted of 24 items in five domains. The scale demonstrated satisfactory construct content, semantic, validity and reliability.

**Conclusions:**

the scale proved to be valid in terms of content and semantics, with a factor structure defined according to the adopted theoretical model and satisfactory psychometric properties.

## RESUMO

**Objetivos:**

elaborar e analisar as propriedades psicométricas da Escala de Risco de Depressão na Gravidez.

**Métodos:**

pesquisa metodológica, em seis etapas: definição empírica do modelo teórico; elaboração dos itens da escala com revisão da literatura; consulta a cinco especialistas profissionais de saúde e 15 gestantes; validação de conteúdo com seis especialistas; pré-teste-validação semântica com 24 gestantes; definição da estrutura fatorial da escala com 350 gestantes; estudo piloto com 100 gestantes, totalizando 489 gestantes e 11 especialistas. Os dados foram analisados pela análise de conteúdo, análise fatorial exploratória, análise multitraço-multimétodo e consistência interna.

**Resultados:**

68 fatores de risco foram identificados para formulação dos itens. A versão final da escala foi composta por 24 itens em cinco domínios. A escala demonstrou validade de conteúdo, semântica, de construto e fidedignidade satisfatória.

**Conclusões:**

a escala mostrou-se válida em conteúdo e semântica, com estrutura fatorial definida segundo modelo teórico adotado e propriedades psicométricas satisfatórias.

## RESUMEN

**Objetivos:**

elaborar y analizar las propiedades psicométricas de la Escala de Riesgo de Depresión en el Embarazo.

**Métodos:**

investigación metodológica, en seis etapas: definición empírica del modelo teórico; elaboración de ítems de escala con revisión de literatura; consulta con cinco profesionales de la salud y 15 mujeres embarazadas; validación de contenido con seis expertos; pre-prueba-validación semántica con 24 gestantes; definición de la estructura factorial de la escala con 350 gestantes; estudio piloto con 100 gestantes, totalizando 489 gestantes y 11 especialistas. Los datos fueron analizados por análisis de contenido, análisis factorial exploratorio, análisis multirrasgo-multimétodo y consistencia interna.

**Resultados:**

se identificaron 68 factores de riesgo para la formulación de los ítems. La versión final de la escala constaba de 24 ítems en cinco dominios. La escala demostró validez de contenido, semántica, de constructo y confiabilidad satisfactoria.

**Conclusiones:**

la escala se mostró válida en contenido y semántica, con estructura factorial definida según el modelo teórico adoptado y propiedades psicométricas satisfactorias.

## INTRODUCTION

Depression is a mental disorder that has spread in recent years, reaching levels that highlight it as a public health concern^(1)^. Worldwide, around 300 million people are affected by depression, being one of the leading causes of disability^(2)^. In pregnancy, depression rates range from 12 to 42% in pregnant women in low- and middle-income countries, according to the World Health Organization (WHO)^(3)^.

Depression in pregnancy, or prenatal depression, has substantial negative consequences that expand from adverse obstetric outcomes^(4)^, such as miscarriages, bleeding^(5)^, negative neonatal outcomes, such as preterm childbirth^(6)^ and potential to influence the child’s cognitive capacity^(5)^, adverse behaviors in pregnant women involving the use of maternal substances and psychiatric hospitalization during pregnancy^(7)^, in addition to the prediction for postpartum depression^(6)^. If left untreated, depression can lead to maternal and child psychological and physical morbidity^(8)^.

Judging by the scope of these repercussions, combined with their severity, the identification of vulnerability to depression and early detection are cornerstones in the prevention and management of this disorder^(9)^.

To screen for depression in pregnancy, as evidenced in the literature, instruments designed to detect depression in the postpartum period have been used, such as the Edinburgh Postpartum Depression Scale (EPDS)^(10)^, as well as general scales for depression, such as the Beck Depression Inventory (BDI)^(11)^, in addition to general instruments for screening mental disorders, such as the Patient Health Questionnaire (PHQ-9)^(12)^.

Although such instruments are screening and assessing depression, they are not specific to be used in pregnancy, and do not cover the risk of developing this disorder, but rather its diagnosis. Thus, in a scenario of health promotion and prevention of obstetric and neonatal diseases, as prenatal care is configured, professionals lack instruments that allow them to assess the risk of depression in pregnancy and, consequently, develop strategies for its prevention.

Although there is a consensus in the literature regarding risk factors for depression in pregnancy, an instrument has not yet been found available to assess the risk of depression among Brazilian women, nor specific to pregnancy. Similarly, an instrument with such characteristics developed in other countries or languages that could be adapted to the Brazilian reality was not found.

This non-existence may be mainly related to difficulties in the construction of instruments. Moreover, when assessing the priority of translation and adaptation studies or instrument development, there is a need to consider the specificity of the study object^(13)^.

In this context, we chose to build an instrument specific to the Brazilian reality, capable of screening the risk of depression in pregnancy, through the detection of risk factors associated with its occurrence, adopting, for construct definition and its factor structure, the theoretical framework of risk of depression in pregnancy and psychometrics^(14)^, respectively.

Considering the above, the instrument in question is not a diagnostic scale. As it is a scale for assessing the risk of developing depression in pregnancy, it becomes essential for nurses in prenatal care, as it supports their clinical practice in identifying the risk of depression in pregnancy, the prevention of this disorder and decision-making about the timely referral to specialized care in psychiatry and other points of the Health Care Network (RAS - *Rede de Atenção à Saúde*), contributing to early intervention and impacting the quality of pregnant women’s mental health. To this impact, the cost reduction for the health system is added, triggered by the use of an easy-to-apply, low-cost screening tool. Initiatives of this nature are more economically interesting than remedying the disorder considering its medicalization and hospitalizations in Tertiary Health Care.

## OBJECTIVES

To elaborate and analyze the Pregnancy Depression Risk Scale (ERDEG - *Escala de Risco de Depressão na Gravidez*) psychometric properties.

## METHODS

### Ethical aspects

Ethical precepts according to Resolution 466 were followed, and the study was approved by the Research Ethics Committees of the *Universidade de São Paulo* at *Escola de Enfermagem de Ribeirão Preto* and by the *Hospital das Clínicas* of the *Faculdade de Medicina de Ribeirão Preto* at the *Universidade de São Paulo*.

### Study design, period, and location

This is a methodological study, which used the assumptions proposed by Pasquali and the DISABKIDS^®^ Group as methodological frameworks^(14-16)^, carried out, from January 12, 2017 to March 15, 2018, at the Usual Risk Prenatal Outpatient Clinic of a public maternity hospital and at the High-Risk Pregnancy Outpatient Clinic of a public university hospital, located in a medium-sized city located in the countryside of the state of São Paulo, Brazil.

### Population and sample; inclusion and exclusion criteria

The population consisted of pregnant women undergoing prenatal care at these outpatient clinics, with 15 pregnant women in step 2; 24 pregnant women in step 4; 350 pregnant women in step 5 and 100 pregnant women in step 6, totaling 489 pregnant women. We included pregnant women aged 18 years or over and who had prenatal care at the outpatient clinics. We excluded those who did not know how to read and/or write, who were unable to communicate verbally and understand without the participation of another person, assessed through observation by the researcher and through consultation with the health team, and not through the application of any assessment instrument. A consecutive non-probabilistic sampling was used, and pregnant women were approached and invited to participate while waiting for prenatal consultation in the outpatient waiting room.

In total, 11 experts participated, five health professionals in step 2 and six health professionals in step 1. We include a health professional in the area of obstetrics and mental health (nurse, physician or psychologist), area of expertise in prenatal care and/or mental health of pregnant women, clinical experience in health care for pregnant women and/or prenatal care with an emphasis on mental health, minimum master’s degree in the area of knowledge of gynecology and obstetrics or psychiatry or psychology. For selection, the *Plataforma Lattes* was used, and recruitment was carried sending invitations via email.

### Study protocol

Data collection was performed by one of the researchers, in a private office, in the outpatient clinics, individually, with each pregnant woman, who received the Informed Consent Form (ICF) on site. Six steps^(14-15)^ were carried out:

1) Theoretical model elaboration: represented the theoretical foundation of the study, in which the theory on the construct^(15)^ was elaborated. These procedures involved the understanding and construct fundamental aspect elaboration, i.e., property, dimensionality, constitutive definition and operational definition, through interpretations and analysis of available references on the problem, including available scales, hypotheses about the object, target audience, objectives and justifications for the relevance of the study and the researchers’ experience.

2) Scale elaboration: comprised the instrument content elaboration in the form of items, based on the risk factors for depression in pregnancy. To define the content and elements to be explored, the identification of risk factors was articulated in three strategies: integrative literature review; consultation with experts in the field through interviews with health professionals in the field of obstetrics and mental health; and consultation with the target population through focus groups with pregnant women^(15)^.

The review was carried out in MEDLINE electronic databases via PubMed, Scopus, CINAHL, PsycINFO and LILACS, between 2012 and 2016. Consultation with the target population was carried out at the High-Risk Pregnancy Outpatient Clinic, with 15 pregnant women with and without a diagnosis of depression, in four groups, with three to four different pregnant women participating in each group. This amount was considered adequate, since an interval of 6 to 15 participants is generally recommended, and when depth of the theme is desired, a smaller group should be chosen^(17)^. Consultation with experts in the area was carried out through individual interviews, guided by a semi-structured script, with five health professionals from the areas of obstetrics and mental health, physicians and psychologists, selected through the *Plataforma Lattes*.

3) Content validity: carried out in order to verify content relevance, clarity and adequacy of all items that compose the scale in relation to the measured construct^(15)^. According to the minimum amount suggested by the literature^(18)^, at this step, six experts participated, being a psychiatrist, a psychologist, two psychiatrist nurses and two obstetric nurses, selected through the *Plataforma Lattes*.

4) Pre-test: semantic validity: performed in order to verify whether the items elaborated were understandable for all members of the target population^(15)^, with the participation of 24 pregnant women selected at the High-Risk Pregnancy Outpatient Clinic.

We used four instruments: a characterization form, filled in by a researcher in the form of an interview; the second version of the scale; a general impressions form, which aims to assess the scale’s general characteristics (importance, number of items, difficulties in answering), answered by all pregnant women; and a specific impressions form, which aims to verify the relevance and understanding of the proposed items, containing three subsets of items (A - items 1 to 11; B - items 12 to 22; C: items 23 to 32).

The general print form and the specific printing form are part of the DISABKIDS^®(16)^ project method, which proposes that each subset of items be answered by at least three pregnant women from each statement. Thus, six pregnant women participated in each subgroup, aiming to cover the lowest stratum and the highest of the target population in relation to education. The lowest stratum of the target population consisted of three pregnant women who completed elementary school. The highest stratum of the target population was composed of three pregnant women who completed high school or more, i.e., completed at least the third year of high school. The mean instrument application time was 20 minutes, this phase being recorded in audio, individually, in a private office.

This step took place in two phases, the first from September 18 to 22, 2017, with the participation of 18 pregnant women, i.e., six in each of the three subgroups. After analyzing the results, the need for changes in some items was identified, for better understanding by the pregnant women, which were changed and submitted to analysis in a second phase, which took place on October 30, 2017, with the participation of 6 pregnant women.

5) Scale factor structure definition: the fifth step resulted in scale factor structure definition, defined according to groups of items that correlate. A total of 350 pregnant women participated in this step, 175 of which were recruited from the High-Risk Pregnancy Outpatient Clinic and 175 from the Usual Risk Prenatal Outpatient Clinic. The total number of participants in this step was defined based on the recommendations so that exploratory factor analysis (EFA) can be carried out, using factor loadings less than or equal to 0.30^(19)^.

6) Pilot study with psychometric properties test: the pilot study analyzed the scale’s psychometric properties, describing construct reliability and validity. A total of 100 pregnant women participated, 50 of which were recruited from the High-Risk Pregnancy Outpatient Clinic and 50 from the Usual Risk Prenatal Outpatient Clinic, meeting the minimum recommendation of 50 participants to carry out the pilot study^(20)^.

### Analysis of results, and statistics

The empirical material resulting from the interviews and focus groups were submitted to thematic content analysis^(21)^. To extract the scale factors, EFA was performed with the estimation method of unbalanced or unweighted least squares (ULS), for considering the ordinal categorical nature of the responses to the items on the dichotomous scale, in addition to the criterion of eigenvalues greater than or equal to one^(22)^. The number of dimensions was established by the criterion of total explained variance and by the extraction performed after the Varimax rotation. To verify EFA adequacy, the Kaiser-Meyer-Olkin (KMO) criterion and Bartlett’ test of sphericity were used^(23)^. For the analysis of psychometric properties, construct validity was assessed through convergent and divergent validity according to multitrait-multimethod analysis (MTMM)^(24)^, using the Multitraid Analysis Program (MAP). Reliability was analyzed through the internal consistency of its items, determined by the Kuder-Richardson coefficient (kr-20)^(20)^. Statistical Package for the Social Sciences (SPSS) was used for statistical analysis.

## RESULTS

### Step 1: theoretical model elaboration

In step 1, construct, according to the depression framework, the risk and risk factor for depression in pregnancy were defined, as reported in a previously published study^(25)^.

### Step 2: scale elaboration

Step 2 began with integrative literature review, in which 3,051 studies were identified, of which 37 comprised the final selection. The results of this review, reported in another publication, showed 34 risk factors, grouped into seven socioeconomic factors, four obstetric and/or maternal factors, seven psychological factors and seventeen psychosocial factors^(26)^.

Consultation with the target population, carried out in the focus groups, showed 10 risk factors, of which four were psychological factors, one was socioeconomic factors, four were obstetric/maternal factors and one was a psychosocial factor, previously published^(27)^.

Consultation with experts in the field revealed 24 risk factors, four of which were socioeconomic factors, three obstetric factors, four psychological factors and thirteen psychosocial factors, reported in another publication^(28)^.

In total, a matrix with 68 risk factors was reached, identified in the three strategies, from which repeated factors were excluded, and 39 were selected. After an analysis performed by the research team, for each risk factor, an operational definition associated with the phenomenon of depression in pregnancy was established to guide the development of each item on the scale. Respecting the recommendations for the elaboration of items^(15)^, we chose to construct them with questions. To measure each item, a new analysis was performed, and dichotomous response alternatives were elaborated, aiming to evidence the presence and absence of the risk factor represented by an item.

After a first draft, new analyzes were carried out to verify the items in relation to similarities, redundancies and their contribution to assess the risk of depression in pregnancy. Subsequently, the items that make up the scale, composition of instructions to respondents, layout and format were selected, culminating in the preliminary version called ERDEG, composed of 32 items.

### Step 3: content validity

According to content assessment, all items presented CVI-I ≥ 0.78, considered excellent, with ten items (31.3%) presenting an index of 0.83, and 22 (68.7%) items showing CVI- I of 1.00. The total set of items presented CVI-S of 0.94, evidencing satisfactory content validity.

As for the scale’s general structure, experts considered the scale to be clear, objective and well-structured. Regarding the items, assessment indicated the maintenance of all of them, however, in 27 of them, changes were suggested, such as: items prepared in a similar way; delete and add new items; add one more response option or modify the proposals; remove, add or replace words or terms used.

The suggestions and observations were assessed by the group of researchers, who played the role of judges, issuing the final opinion. Some suggestions were accepted, aiming at a better understanding, and grammatical modifications were made with removal or addition of words or terms, substitution of words or negative terms, as well as considered difficult for the understanding of pregnant women with less education. The restructured scale was sent back to the same experts, with unanimity in agreement on the adjustments made. Thus, the second version of ERDEG was elaborated.

### Step 4: pre-test - semantic validity

Due to semantic validity, the scale was well accepted and considered easy to understand by pregnant women. Among the participants, 66.7% (16) considered the scale a “very good” instrument; 95.8% (23) considered it important to assess the risk of depression in pregnancy; 91.6% (22) interpreted the answers as “easy to understand”; and for all pregnant women, there was no need for change or addition. Four pregnant women (9.6%) expressed the desire not to want to answer some items, an attitude justified by specific questions and motivated by specific reasons, mentioning the questionnaire size and the difficulty of understanding.

In the analysis of the specific form regarding importance, three items on the scale were not considered important by a pregnant woman. For the analysis of the difficulty of understanding the items and the response options as clear, a percentage of 80% or more was considered ideal, i.e., the understanding of an item was considered adequate when, at least, 80% of pregnant women had no difficulty in understanding it. The same occurred for the response options for the item, which were considered ideal, when they were clear to at least 80% of pregnant women. In two items, 66.6% of pregnant women had no difficulty in understanding it, showing a percentage below the expected of 80%, indicating the need to reformulate an item. Regarding the response options, one item was clear for 50% of pregnant women, and two other items, the rate was 66.6% of participants, evidencing the need to reformulate the response options.

As for the meaning, all items were understood by pregnant women, but suggestions for reformulation in participants’ own words were pointed out in 15 items. It was noted that some pregnant women suggested reformulation, even though they understood the item and its answers. Thus, the suggestion was only accepted, and the item was reformulated, when the percentage of 80% was not reached for understanding the item and its answers, which occurred in three items that were later submitted again to the semantic validity process, seeking to verify whether the changes were relevant. In the reassessment, all the pregnant women considered two reformulated items important, and, for one item, the importance was attributed by most of them (83.3%). No pregnant woman had difficulty understanding the items and their response options; all understood the meaning of all items; and none suggested further modifications.

After consensus in analysis, the third version of the scale was elaborated.

### Step 5: scale factor structure definition

The KMO test provided a value of 0.739, considered median, which indicated that the factor analysis was appropriate to be performed, since it was between the reference values 0.5 and 1^(18)^. Bartlett’s test of sphericity presented p=0.0001 and χ2(990)=2170.658, which allowed rejecting the null hypothesis that the data matrix is similar to an identity matrix, and confirmed the method analysis use for the data collected^(22)^.

The EFA revealed an initial factorial solution of eleven dimensions, opting, after analysis, according to the theoretical framework adopted, for the extraction of 5 dimensions, which together explained 37.6% of the total variation (Table 1).

**Table 1:** Pregnancy Depression Risk Scale factor loading matrix, Ribeirão Preto, São Paulo, Brazil, 2018

Scale items		Dimension or factor				
		1	2	3	4	5
1^*^	How old are you?	-0.07	-0.06	-0.21	-0.11	0.11
2^*^	What is your education level?	-0.08	0.02	0.00	0.24	0.19
3	What is your current marital status?	-0.06	0.55^†^	-0.13	0.20	-0.13
4	How do you define your current family income?	0.14	0.01	0.10	0.69^†^	0.03
5	Considering the people who live with you, what is the head of the family’s labor situation?	0.02	0.10	-0.09	0.50†	0.04
6	How do you consider your social situation?	0.10	0.07	0.03	0.44^†^	0.04
7	In this pregnancy, do you use illegal drugs?	0.04	-0.03	-0.06	-0.00	0.40^†^
8	In this pregnancy, do you drink alcohol?	0.01	0.03	-0.00	0.07	0.52^†^
9	In this pregnancy, do you smoke cigarettes?	0.03	0.00	-0.01	0.04	0.51^†^
10^*^	In the last 12 months, have you experienced a remarkable negative event in your life?	0.29	0.16	0.20	0.01	0.01
11	Did you suffer any kind of violence before this pregnancy?	0.27	0.35^†^	0.19	0.02	0.18
12	Did you suffer any kind of violence in this pregnancy?	0.10	0.47^†^	0.11	0.00	0.27
13^*^	Do you face arguments with your partner in this pregnancy?	0.12	0.27	0.08	0.21	0.24
14	How do you define your relationships with people?	0.08	0.46^†^	0.02	0.02	0.08
15^*^	Do you have any support in difficult times?	0.11	0.25	0.02	-0.01	-0.01
16^*^	Do you have a religion?	-0.01	0.05	-0.10	0.03	0.22
17	Do you have the support of your child’s father in this pregnancy?	0.01	0.66^†^	-0.04	0.25	-0.10
18^*^	Do you have a religion?	0.22	0.21	0.05	0.12	0.20
19	In this pregnancy, how do you define your mood most of the time?	0.35	0.39^†^	0.15	0.18	0.22
20	In this pregnancy, have you been worried, more than usual, to the point of harming you?	0.58^†^	0.07	0.30	0.16	-0.03
21	In this pregnancy, do you experience stressful situations that you consider to be harming you?	0.47^†^	0.25	0.14	0.09	0.11
22	Do you have pregnancy-related fears?	0.49^†^	-0.04	0.21	0.10	0.12
23	Do you want this pregnancy?	0.06	0.46^†^	0.12	0.04	0.01
24	Have you had depression at any point in your life?	0.38^†^	0.28	0.03	0.05	-0.01
25	Do you have cases of depression in your family?	0.33^†^	0.12	-0.02	-0.10	-0.05
26	In this pregnancy, do you feel anxious, more than usual, to the point of interfering with your daily life?	0.58^†^	-0.10	0.00	0.13	0.09
27	Do you have any health problem?	0.16	0.08	0.59^†^	-0.00	-0.02
28	How do you define your ability to adapt to new life situations?	0.40^†^	0.10	0.09	0.16	-0.04
29^*^	Was this pregnancy planned?	0.09	0.12	0.02	0.20	0.02
30	Is your pregnancy at risk?	0.12	-0.02	0.78^†^	0.00	-0.07
31	In any previous pregnancies did you face complications?	0.34^†^	-0.01	0.20	-0.01	-0.04
32	In this pregnancy, do you face complications?	0.24	0.11	0.60^†^	-0.01	-0.06

* *Item eliminated from the scale according to factor loading criterion ≤ 0.30; †Highest factor load presented by the item. This scale was freely translated.*

As explained, 8 items that presented factor loadings less than or equal to 0.30^(19)^ were excluded. Subsequently, a second EFA was performed in order to verify the explained variance of this model with 24 items and five dimensions, which corresponded to 46.2%, as described in Table 2.

**Table 2:** Explained variance of scale dimensions or factors after the second exploratory factor analysis, Ribeirão Preto, São Paulo, Brazil, 2017

Dimension or factor		Initial eigenvalues			
	Total	% variance	% accumulated	Total	% variance
1	4.192	17.465	17.465	4.192	17.465
2	2.246	9.360	26.824	2.246	9.360
3	1.718	7.159	33.984	1.718	7.159
4	1.576	6.568	40.552	1.576	6.568
5	1.356	5.649	46.202	1.356	5.649
6	1.104	4.602	50.803		
7	1.050	4.376	55.179		
8	.962	4.010	59.189		
9	.929	3.869	63.058		
10	.848	3.532	66.590		
11	.826	3.441	70.031		
12	.796	3.315	73.347		
13	.734	3.057	76.404		
14	.690	2.876	79.280		
15	.676	2.815	82.095		
16	.597	2.488	84.583		
17	.581	2.421	87.004		
18	.556	2.318	89.322		
19	.536	2.232	91.555		
20	.476	1.983	93.538		
21	.435	1.811	95.349		
22	.415	1.730	97.079		
23	.379	1.578	98.657		
24	.322	1.343	100.000		

Based on the changes, we developed the final version of the scale, consisting of 5 dimensions and 24 items, arranged in the form of an interrogative sentence, with responses listed in two categories, in order to preserve the presence and absence of the risk factor contemplated by the item, as presented below.

The scale’s 5 dimensions were psychic, psychosocial, maternal health, socioeconomic and psychoactive substances, which correspond to the risk to which the item refers. Among the items, only two (items 19 and 31) were allocated to a dimension different from the risk to which they refer. However, they were kept in the dimension proposed by EFA, since they presented a greater factor loading in these dimensions than in the dimension corresponding to risk. Item 19 had a factor loading of 0.35 in the dimension corresponding to psychic risk and 0.39 in the psychosocial dimension, to which it was allocated. Item 31 presented a factor loading of 0.20 in the dimension corresponding to obstetric risk and 0.34 in the psychic dimension, in which it was allocated.

In addition, one dimension brought together three items, separately, that relate to psychosocial risk. These were kept in this dimension because, although the statistical model did not group them together with the other items that refer to psychosocial risk, the grouping incorporated similar items covering the same theme, related to the use of psychoactive substances such as alcohol and drugs.

### Step 6 - pilot study with psychometric properties test

For convergent validity analysis, values of Pearson’s linear correlation coefficients greater than or equal to 0.30 were considered satisfactory. This criterion was met by 2 of the 3 items of “socioeconomic” and “psychoactive substances” dimensions, by all 7 items of the “psychosocial” dimension, by 6 of the 8 items of the “psychic” dimension and by all 3 items that composes the “maternal health” dimension. Among the four items that did not meet the established criteria (items 2, 5, 20 and 21), it is noteworthy that the correlation values of items 5 and 20, although they are below the expected (0.30), are close to if the established criterion, given that they have values of 0.28 (Table 3).

**Table 3:** Pearson’s correlation coefficient values between the items and each of the Pregnancy Depression Risk Scale dimensions according to multitrait-multimethod, Ribeirão Preto, São Paulo, Brazil, 2018

Item	Dimension					
	Socioeconomic	Psychoactive substances	Psychosocial	Psychic	Maternal health	Total
1	0.32^*^	0.05	0.21	0.22	0.17	0.31
2	0.26^*^	0.08	0.11	0.11	0.08	0.18
3	0.38^*^	-0.10	0.06	0.14	-0.06	0.13
4	0.05	0.34^*^	0.02	0.15	0.08	0.17
5	-0.02	0.28^*^	0.07	-0.03	-0.22	-0.05
6	0.04	0.43^*^	0.07	0.12	0.01	0.14
7	0.13	0.04	0.53^*^	0.28	0.16	0.39
8	0.14	0.18	0.39^*^	0.32	0.20	0.42
9	-0.01	-0.05	0.49^*^	0.19	0.17	0.29
10	0.18	-0.07	0.63^*^	0.13	0.20	0.33
11	0.11	0.07	0.61^*^	0.25	0.08	0.35
12	0.17	0.25	0.32^*^	0.12	0.11	0.27
13	0.12	-0.02	0.41^*^	0.18	0.15	0.29
14	0.19	0.13	0.23	0.54^*^	0.19	0.49
15	0.38	0.06	0.33	0.53^*^	0.20	0.56
16	0.06	0.01	0.19	0.38^*^	0.23	0.36
17	0.06	0.09	0.31	0.41^*^	0.32	0.47
18	0.00	-0.13	0.18	0.30^*^	0.04	0.21
19	0.18	0.03	0.06	0.40^*^	0.11	0.31
20	0.08	0.28	0.05	0.28^*^	0.06	0.25
21	0.09	0.05	0.24	0.27^*^	0.25	0.34
22	-0.01	-0.12	0.10	0.24	0.52^*^	0.31
23	0.15	-0.02	0.24	0.27	0.81^*^	0.48
24	-0.04	0.28	0.31	0.88	0.53^*^	0.53

* *Pearson’s correlation coefficient of an item with its respective dimension.*

**Table 4:** Multitrait Analysis Program results for Pregnancy Depression Risk Scale scores, Ribeirão Preto, São Paulo, Brazil, 2018

	Dimension					
	Socio economic	Psychoactive substances	Psychosocial	Psychic	Maternal health	Total
	Nº items (%)	Nº items (%)	Nº items (%)	Nº items (%)	Nº items (%)	Nº items (%)
-2	0 (0)	0 (0)	0 (0)	0 (0)	0 (0)	0 (0)
-1	0 (0)	0 (0)	0 (0)	1 (3.1)	0 (0)	1 (1)
1	7 (58.3)	2 (16.7)	5 (17.9)	23 (82.1)	28 (100)	25 (26)
2	5 (41.7)	10 (83.)	23 (82.1)	20 (62.5)	12 (100)	70 (72.9)
1+2	12 (100)	12 (100)	28 (100)	31 (96.9)	12 (100)	95 (99)
Adjustment	100%					

In the divergent validity, the MAP showed satisfactory results, since four dimensions presented values of 100% of fit and one dimension had values above 90% of fit, considering the criterion of being as close as possible to 100%. Moreover, the scale as a whole showed an adjustment of 99%.

The Kuder-Richardson coefficient (kr-20) value, used for reliability analysis, was compared with the threshold conventionally considered adequate (kr≥0.70)^(29)^. The internal consistency of three dimensions (psychosocial, psychic, maternal health) and the total scale was satisfactory, considering that kr-20 values were above expectations. However, the “socioeconomic” and “psychoactive substances” dimensions presented kr-20 values below the desired (Table 5).

**Table 5:** Kuder-Richardson coefficient (kr-20) for the Pregnancy Depression Risk Scale dimensions, Ribeirão Preto, São Paulo, Brazil, 2018

Dimension	Nº items	Kr-20
Socioeconomic	3	0.49
Psychoactive substances	3	0.54
Psychosocial	7	0.76
Psychic	8	0.70
Maternal health	3	0.86
Total ERDEG	24	0.77

Given the above, the satisfactory results expressed good psychometric properties of the ERDEG.

## DISCUSSION

In this ERDEG development study, we adopted a description of the steps, with the option of explaining the actions carried out in a logical sequence, regardless of the denomination or conceptualization attributed to the phases, such as steps or types of validity, similar to a previous study^(13)^.

In this process of scale development, the strategies adopted to support item elaboration, consisting of a literature review, consultation with the target population and consultation with experts in the area, allowed identifying the data in its completeness and that best represented the construct of interest. This strategy, which covers all the possibilities proposed by the methodology used^(15)^, is corroborated by a Norwegian study, which reports the development of an instrument aimed at diabetes care, which was based on a literature review, consultation with the target population through interviews with parents and children and consultations with groups of experts^(30)^. In a Japanese study, the strategies used by the authors differ from this study, when they only consulted the target population using questionnaires^(31)^.

When comparing the scale construction process with other studies of instrument development and validity, it was observed the usual use of expert judgment as a strategy to analyze item content and format. This procedure underlies the content validity used in this study, which was widely performed by other researchers in the development of their instruments in Norway^(30)^ and Netherlands^(32)^. It is noteworthy the diverse composition of expert committee for content validity with different professional categories that, similar to a previous study^(30)^, allowed a broad and in-depth assessment, with pertinent and complementary observations, which culminated in a satisfactory content validity evidenced by Content Validity Index of the scale of 0.94. This index is higher than that shown in instruments developed in India^(31)^ and Iran^(33)^, and similar to the index found in an instrument developed in the United States^(34)^.

Semantic validity, in turn, demonstrated the easy understanding of the scale by pregnant women, evidencing the importance of this procedure in the development of instruments, corroborated by other authors^(35)^. Given the above, it is evident that the scale developed presented content validity and semantic evidence.

The scale dimension definition highlighted the model composed of five dimensions or factors, which gave it a multidimensional characteristic. Equivalent results were observed in the development of other instruments, such as the Diabetes Care Questionnaire (PEQ-DC)^(30)^ and the Patient experiences questionnaire for interdisciplinary treatment for substance dependence (PEQ-ITSD)^(36)^, both prepared in Norway.

It is noteworthy that the model, composed of five dimensions, grouped the items into homonymous dimensions to the corresponding risk in 22 of the 24 items on the scale, significantly approaching the basis on risk factors for depression in pregnancy.

It is also worth mentioning that the choice of the number of dimensions or factors is a challenge, corroborated by other authors^(37-38)^, for which the balance between parsimony and information significance is sought, since the overestimation of the number of factors can lead to the production of an exaggerated number of constructs, due to the number of excessive and superfluous dimensions with little explanatory power. On the other hand, a very small number of dimensions can culminate in significant loss of information^(37-38)^.

A pilot study showed that, in convergent validity analysis, in all dimensions, most items presented satisfactory correlations with their dimensions. In divergent validity, the satisfactory results expressed by the total fit in four of the five dimensions, and by values close to this for one dimension, denoted a satisfactory correlation, in most cases between the item and its dimension than between it and any other dimension. The same was also observed for the scale in general. This demonstrates that the proposed model confirmed the correct allocation of items in the appropriate dimension, initially defined.

Regarding reliability, in general, kr-20 was satisfactory for the total scale, which denotes good internal consistency of its items. Three dimensions presented satisfactory results for internal validity with coefficient values above expected. Two dimensions presented values below the expected, which could be associated with the small number of items in their conformation, considering that they have three items each and that coefficient values are strongly influenced by the number of items in the measurement instrument, so that the small number of items per dimension of an instrument can decrease coefficient values, affecting internal consistency^(39)^. However, individual analysis of this statistic cannot be considered for decision making in relation to dimensions, since other statistics presented satisfactory values, such as convergent and divergent validity.

The satisfactory results obtained in the pilot study supported the continuity of this study and allowed carrying out a field study with definition of the final psychometric properties, which will be presented in another publication.

### Study limitations

We sought to avoid selection bias, composing the sample with pregnant women with usual and high risk of pregnancy. However, it was composed of a high number of pregnant women, mostly from the same region.

### Contributions to obstetrical and mental health nursing

The development of an unprecedented and specific scale for screening the risk of depression among Brazilian pregnant women responds to a need and has the potential to support: clinical practice of nurses and other health professionals in prenatal care; qualification of care for pregnant women; and improvement of health care in the Brazilian scenario, resulting in better maternal and child indicators.

The development of a technology explains how much the elaboration of an instrument contributes to public health, especially for obstetric nursing, with emphasis on pregnant women’s mental health. In addition to this, building an instrument capable of measuring the risk of depression in pregnancy can confirm problems in instruments adapted or not specific for the use in question.

## CONCLUSIONS

The results of this study demonstrate that the ERDEG is a clear, objective, well-structured instrument that is easily understood by pregnant women, with a factor structure defined according to the theoretical model adopted and with satisfactory psychometric properties, expressed by construct validity and reliability.

The development of a specific depression risk screening scale for use in pregnancy among Brazilian women is unprecedented. The ERDEG is not a diagnostic scale and its use does not replace a specialized clinical assessment in mental health. Therefore, the scale developed allows tracing the scenario of vulnerability of pregnant women to depression, being able to contribute with nurses in prenatal care in assertive decision making, in prevention and promotion of mental health, mainly in the usual risk prenatal care developed in Primary Health Care (PHC), qualifying health care and improving nursing practices, benefiting the profession.
